# Testing three seismic hazard models for Italy via multi-site observations

**DOI:** 10.1371/journal.pone.0284909

**Published:** 2023-04-27

**Authors:** Iunio Iervolino, Eugenio Chioccarelli, Pasquale Cito

**Affiliations:** 1 Dipartimento di Strutture per l’Ingegneria e l’Architettura, Università degli Studi di Napoli Federico II, Naples, Italy; 2 IUSS–Scuola Universitaria Superiore di Pavia, Pavia, Italy; 3 Dipartimento di Ingegneria Civile, dell’Energia, dell’Ambiente e dei Materiali, Università degli Studi Mediterranea di Reggio Calabria, Via Zehender, Reggio Calabria, Italy; Ud’A: Universita degli Studi Gabriele d’Annunzio Chieti Pescara, ITALY

## Abstract

Probabilistic seismic hazard analysis (PSHA) is widely employed worldwide as the rational way to quantify the uncertainty associated to earthquake occurrence and effects. When PSHA is carried out for a whole country, its results are typically expressed in the form of maps of ground motion intensities that all have the same exceedance return period. Classical PSHA relies on data that continuously increase due to instrumental seismic monitoring, and on models that continuously evolve with the knowledge on each of its many aspects. Therefore, it can happen that different, equally legitimate, hazard maps for the same region can show apparently irreconcilable differences, sparking public debate. This situation is currently ongoing in Italy, where the process of governmental enforcement of a new hazard map is delayed. The discussion is complicated by the fact that the events of interest to hazard assessment are intentionally rare at any of the sites the maps refer to, thus impeding empirical validation at any specific site. The presented study, pursuing a regional approach instead, overcoming the issues of site specific PSHA validation, evaluated three different authoritative PSHA studies for Italy. Formal tests were performed directly testing the output of PSHA, that is probabilistic predictions, against the observed ground shaking exceedance frequencies, obtained from about fifty years of continuous monitoring of seismic activities across the country. The bulk of analyses reveals that, apparently alternative hazard maps are, in fact, hardly distinguishable in the light of observations.

## Introduction

### Countrywide PSHA essentials

There is consensus on probabilistic seismic hazard analysis or PSHA [1, 2] being the only way to quantitatively deal with the large uncertainty (i.e., lack of knowledge) about earthquake occurrence and effects. Given a measure of ground motion intensity, and one of its possible values, PSHA enables calculating the annual frequency, or equivalently the return period, of exceedance of such a value, at a site of interest.

PSHA is primarily used for quantitative seismic risk assessment of critical or strategic civil infrastructure. A related application is to define the seismic actions for structural design or assessment, with the aim of controlling the structural safety and/or for urban planning. In this case, PSHA is carried out at a national level and its results are typically expressed in the form of maps providing the ground motion intensities at the sites, which are all exceeded with the same return period [3].

Classical countrywide PSHA models rely on three main datasets and component models: (1) a catalog with location, date, and magnitude of past earthquakes, either historically documented or instrumentally recorded (or equivalent information as it can be inferred from other sources, such as geodetic data [4]); (2) a seismic source model, for example, as geologically homogeneous zones where earthquakes can occur (possibly accompanied by individual faults, if available); (3) a ground motion model (GMM), calibrated on recorded ground motion data, to probabilistically relate the earthquake magnitude and location, and other covariates, to the shaking at the site of interest. Because research nowadays produces several alternative models to address each of these components, PSHA is often executed combining them in the form of logic trees [5], the complexity of which can impair the reproducibility of the analysis.

In fact, although earthquakes occur as clustered both in time and space [6, 7] (commonly referred to as earthquake sequences or swarms), PSHA only contemplates the largest magnitude event of each cluster, the so called-mainshocks, so that it avoids some catalog incompleteness issues [8], and enables more easily associating a return period to the exceedance of ground motion intensity thresholds. Moreover, only the mainshocks above a minimum magnitude of interest are considered.

Due to the rapid evolution of seismic monitoring networks, which leads to an exponentially increase in the number of recorded earthquakes [9] and the continuous research results produced in the field [10]. national hazard studies are updated relatively frequently, that is every few years is some cases [11].

### Current issues

The issue of empirical validation of probabilistic seismic hazard maps is a major one, given the safety- and economy-related impact they have in the country where they are enforced. However, the ground motion intensities of interest for engineering and risk management applications are those associated with relatively long return periods according to PSHA. This is because, for example, when seismic design is of concern, structural safety must be warranted with respect to relatively rare events. Therefore, it is typical that the return periods inferred from PSHA cannot be evaluated against observations at any site in the region where PSHA has been carried out, as the available seismic history is most likely largely insufficient [12, 13].

Moreover, despite hazard maps are defined in terms of return period of exceedance of ground motion intensity values, they are often questioned with respect to possible fallacies in the analysis whenever such exceedance occurs. The arguments being that these exceedances are too frequent [14], even if research has clarified that it is often an illusion and that, when moderate-to-high magnitude earthquakes occur, it is well expected by PSHA that the intensities from hazard maps are exceeded in an area around the epicenter, the size of which is depending on the event magnitude and the return period the map refers to [15, 16]. Critiques also stem from the fact that earthquake occurrence on faults may follow a recurrence pattern that departs from the memoryless process assumption taken in classical PSHA [17], although this issue has been known since the original developments of PSHA, and it has been shown not to be generally a limitation, especially in the case of large source zones [18].

Finally, it might be that different authoritative hazard maps, developed by different contemporary studies, are available for the same area, so that superiority cannot solely be established based on one being more up to date than another. In addition, the complex logic trees, which may feature thousands of branches, used to account for the various visions on the models to be considered in PSHA, impair the possibility to investigate in-depth these studies.

These issues can generate mistrust in PSHA, not only in the public, but also in part of the scientific community and, most importantly, in the regulators and authorities enforcing hazard models. This is a situation possibly currently occurring in Italy, where the official seismic hazard map, MPS04 hereafter [19], used for seismic design, dates about twenty years ago. Recently, an update of the national building code has been accompanied by the development of a new hazard map, indicated hereafter as MPS19 [20], and that is not yet enforced because of a long and controversial approval process. A third recent model, which can be indicated as ESHM20 [21], developed for the whole Europe, has been chosen to accompany the second generation of Eurocode 8 [22], the continental building standard. The hazard maps from these PSHA models show differences that are large according to some, especially in some areas of the country, and are causing significant debate along some of the lines discussed above, as recently reported [23].

### This study

Italy is provided with a now very dense seismic monitoring network, featuring several hundreds of recording stations [24]. Systematic monitoring of earthquake activity goes on since the early Seventies and it is possible to find several stations that have been continuously operating most of the time ever since.

Based on empirical data from seismic monitoring, the study herein has evaluated the output of three available hazard maps, formally testing whether the observed occurrences of earthquakes, and the consequent ground motion intensity exceedances, are compatible with what predicted from the PSHA models.

Because it is almost impossible to gather sufficient data to undertake this task at any specific site in the region the map refers to, as it would require thousands of years of continuous recording, the tests were carried out pooling the data from the recording stations that have operated the longest in the country. This is because, if the models used to develop the tested map are used to obtain the probability distribution of the total number of exceedances at several sites over time, then data from much shorter observation periods can be sufficient.

Because hazard maps collect PSHA results developed one site at a time, they do not account for the stochastic dependence of intensities at different sites affected by the same earthquake. Therefore, testing required running multi-site hazard analyses [25], described in the following and which imply that the results of the tests do not apply to any specific site, yet rather generally to the national scale the map refers to.

The remaining paper proceeds as follows. In the Materials and methods section, the PSHA models at comparison and (a subset of) hazard maps being tested are introduced; then, the illustration of empirical data is followed by the description of the analyses needed to develop the statistical tests. The outcomes of such tests are discussed in the Results section. The main conclusions that can be drawn from the study close the paper.

## Materials and methods

### Three authoritative seismic hazard maps for Italy

MPS04 is based on a logic tree with sixteen branches; however, herein the branch named 921 is considered. This is because it is the branch considered to best represent the median result of the logic tree [26]. According to branch 921, for each of the thirty-six seismic zones constituting the source model [27], the seismicity is defined in terms of *activity rates*, that is, annual rates of earthquakes associated to (surface wave) magnitude (M) bins with width equal to 0.3 magnitude units. The lowest magnitude bin is centered at M4.3 for all zones (apart from the Etna’s volcanic area, where minimum magnitude is lowered to M3.7), whereas the central value of the largest magnitude bin is zone dependent and can be up to M7.3 [28]. The GMM is that of Ambraseys et al. [29], which is combined with the correction factors proposed by Bommer et al. [30], to account for the predominant style-of-faulting of each source.

MPS19 has been developed about twenty years after MPS04. It is based on ninety-four alternative source models and GMMs. The logic tree of MPS19 counts about six-hundred branches, and therefore its full implementation in PSHA can be challenging. However, precisely with the purpose of ease of implementation, a weighted average grid-seismicity source model was developed [31]. It features eleven-thousand point-sources for which seismicity is defined by activity rates for (moment) magnitude bins 0.1 wide. The minimum magnitude is M4.5, the maximum is M9.0 for about 85% of sources, and it is M8.3 in the rest. For each point, a probability distribution for the style-of-faulting is also defined. This source model, coupled with the GMM of Bindi et al. [32], which is the one with the largest weight, defines the second hazard model considered.

The 2020 European Seismic Hazard Model or ESHM20, considered an update of ESHM13 [33], was developed to provide a homogenous seismic hazard assessment of the continent region, without country-border issues. For the hazard assessment in the regions characterized by shallow crustal earthquakes, including Italy, ESHM20 adopts a logic tree consisting of two main branches, one for the area source models and the other for active faults with background seismicity; further branches account for different magnitude distributions and seismicity parameters. Minimum magnitude is 4.5, whereas the largest value can be as high as 8.4. The GMM includes alternative adjustment factors applied to quantify the uncertainties associated to different regions; i.e., the so-called *backbone* approach. The GMM also accounts for the epistemic uncertainty by means of a logic tree [34]. The analyses herein are based on the two branches for the source models and one branch for the GMM, that is the one with the largest weight. This set of models is claimed to provide PSHA results approximating those representing the mean of the whole ESHM20 logic tree implementation (L. Danciu, personal communication, 2021).

**Fig 1:**
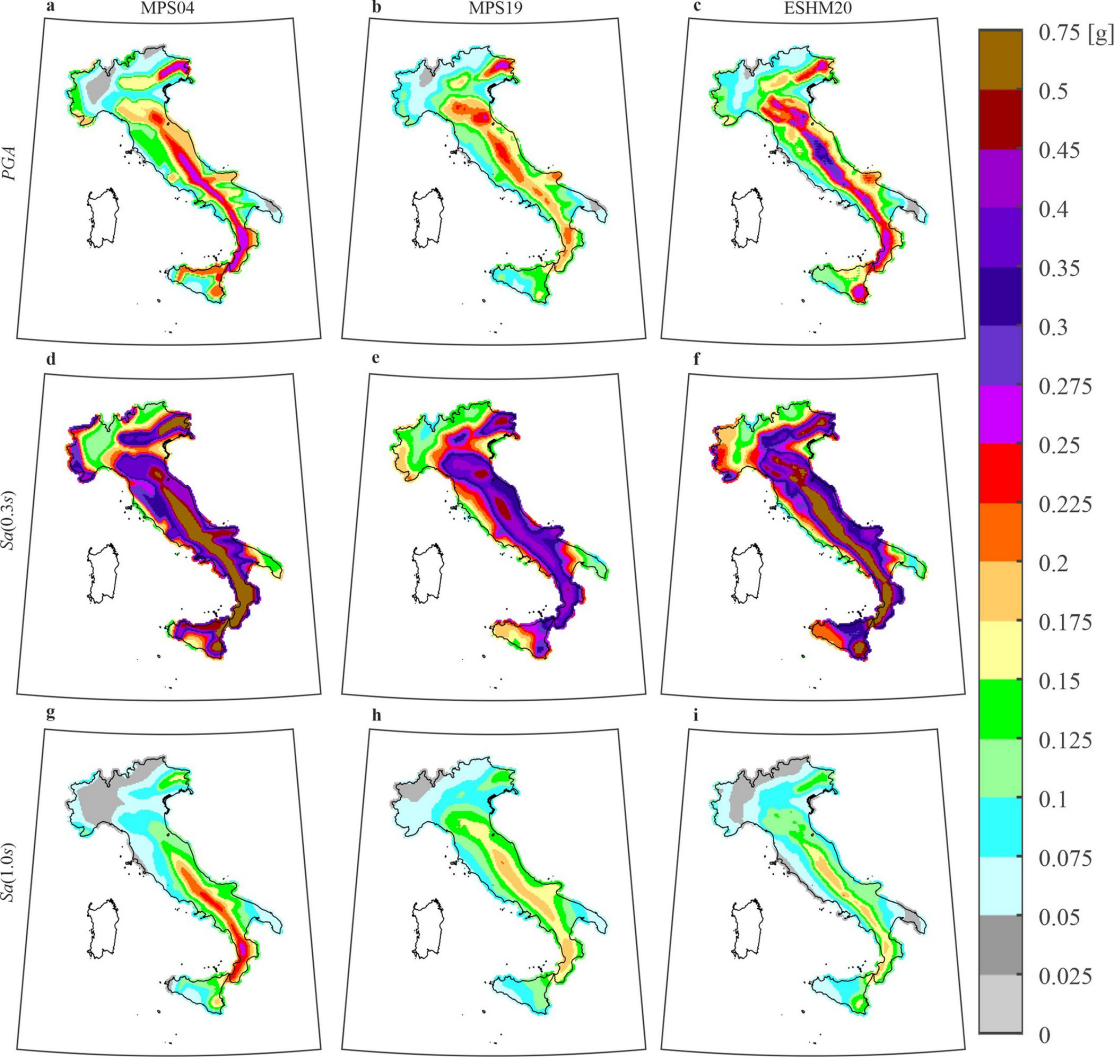
*T*_*r*_ = 475*yr* hazard maps on rock according to three authoritative models for three intensity measures. The panels in the same columns refer to the same model: (a), (d), (g) are from MPS04; (b), (e), (h) are from MPS19; (c), (f), (i) are from ESHM20. The panels in the same row refer to the same ***IM***: (a), (b), (c) are ***PGA***; (d), (e), (f) are ***Sa***(**0.3*s***); (g), (h), (i) are ***Sa***(**1.0*s***). The maps were generated using the Matlab mapping toolbox version 4.10 (https://it.mathworks.com/products/mapping.html).

For the purposes of this study, twelve hazard maps on reference rock site conditions (A class according to Eurocode 8) are derived from each hazard model; they refer to four exceedance return periods, *T*_*r*_, equal to 50, 475, 975, and 2475 years, and three ground motion intensity measures (*IMs*), that are the peak ground acceleration, *PGA*, and the pseudo-spectral acceleration associated to a vibration period equal to 0.3s and 1.0s, indicated as *Sa*(0.3*s*) and *Sa*(1.0*s*), respectively. A subset of the resulting maps, that is, those referring to *T*_*r*_ = 475*yr*, for the tree *IMs*, is given in Fig 1. For comparability, because the GMMs associated to the three hazard models refer to different values of horizontal components of shaking, the MPS19 and ESHM20 maps were converted into the largest component according to the model of Beyer and Bommer, that is the intensity measure of MPS04 [35]. This is also consistent with the empirical ground motion data used in the study, which consider the largest horizontal component recorded at the station sites (see next section).

Comparing the maps, it can be seen that seismic hazard can differ significantly in some areas. Referring to *PGA*, it appears that ESHM20 generally provides the largest values, whereas the values associated to MPS19 are the lowest, and those associated to MPS04 are intermediate. However, there are limited areas (e.g., western Sicily and the western coasts of central Italy) in which MPS04 provides the largest *PGA* and ESHM20 the lowest. If *Sa*(0.3*s*) is considered, MPS04 and ESHM20 appear in relatively good accordance while MPS19 provides the lowest values. When *Sa*(1.0*s*) is of interest, considering southern Italy, the largest values are associated to MPS04 and the lowest are computed via ESHM20; on the other hand, referring to the northern Italy, the lowest values are associated to MPS04. Comparing also the maps associated to the other return periods (not shown here for the sake of brevity), for example considering the site of L’Aquila, generally representative of high hazard in Italy, the relative differences between two hazard maps, for the same return period and *IM*, can be larger than 35%. This kind of comparisons are stimulating the debate around the model that should be enforced for seismic design.

### Empirical data

The largest seismic monitoring network in Italy is the *Rete Accelerometrica Nazionale* (RAN) and, in its current configuration, is constituted by more than six-hundreds stations. All recorded waveforms from RAN, together with those from other accelerometric networks, are collected and made available by *Istituto Nazionale di Geofisica e Vulcanologia* (INGV) in online repositories; i.e., the Italian Accelerometric Archive or ITACA (https://itaca.mi.ingv.it/ItacaNet_32/#/home).

INGV has provided the authors with the recording stations of the RAN that worked the longest, which are 143 and collected data from 1973 to 2019 overall (L. Luzi, personal communication, 2021). For most stations, the operating time, that is, the time interval between the installation and removal year is between 40 and 45 years, and for some of them approaches 50 years (see next section). For each of the 143 stations, and for each of the *IMs* considered in the study, the maximum recorded value, among those associated to the two horizontal components, per year, was also available.

Using the data in ITACA, that contains the waveforms of the recorded earthquakes with magnitude larger than the minimum considered by the models, it was verified that: (i) the maximum recorded *IM* per year is caused by a mainshock, according with the declustering analysis of the *Catalogo Parametrico dei Terremoti Italiani* or CPTI15 [36] (see S1 Table, in which mainshocks up to 2019 are considered; A. Rovida, personal communication, 2019); (ii) in the same year of the maximum value available, no other mainshocks were recorded by the same station. The former check was needed to avoid counting *IM* exceedances due to earthquakes that are not contemplated by PSHA, the latter was to integrate, if needed, the available information with other exceedances in one year, caused by earthquakes considered to be mainshocks by seismologists.

**Fig 2:**
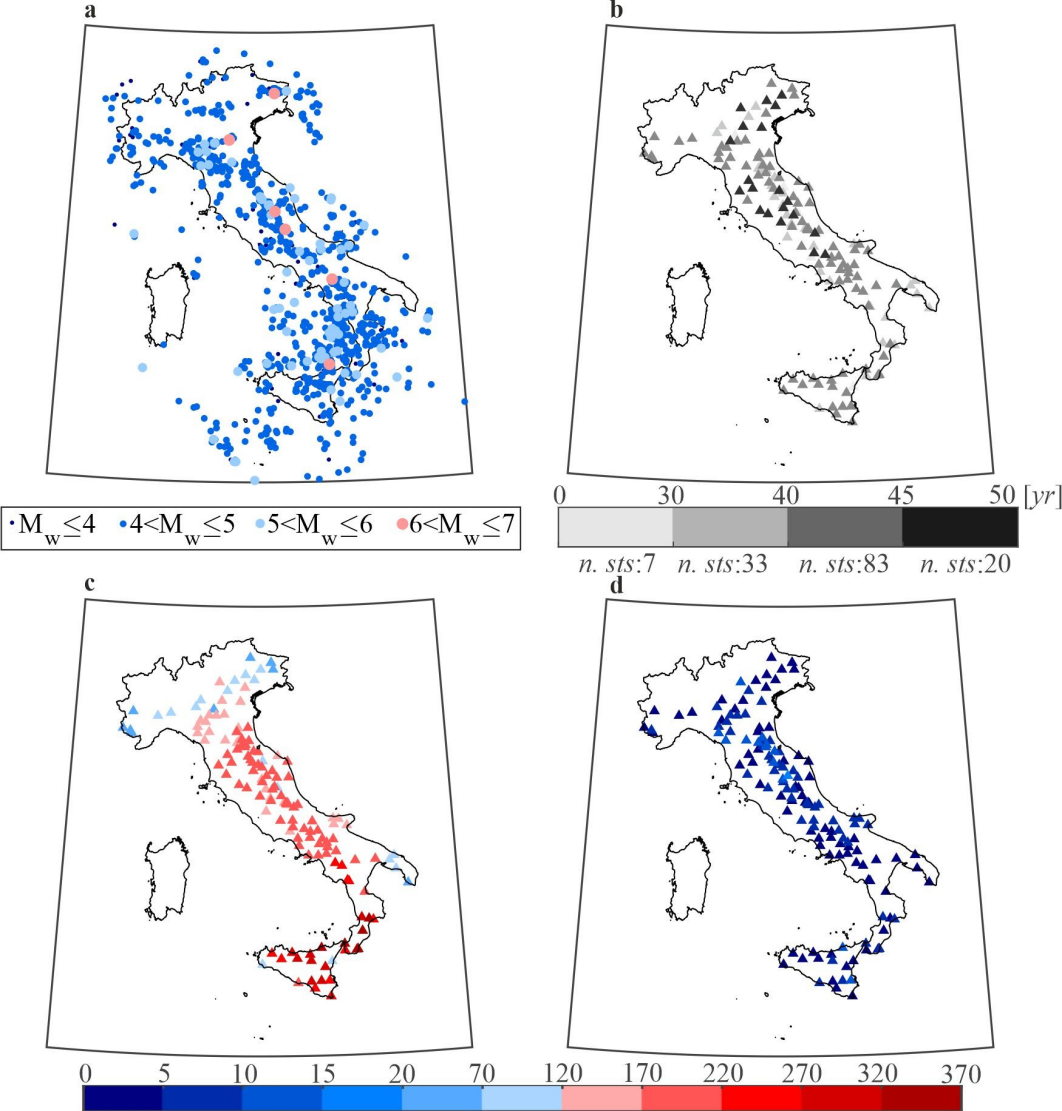
Earthquakes and stations considered in the study. (a) M3.7+ mainshocks from 1973 to 2019 according to CPTI15. (b) Considered stations and their operating time (in terms of number of years). (c) Number of mainshocks within 200 km per station during operating time according to CPTI15. (d) number of recorded mainshocks for each station. The maps were generated using the Matlab mapping toolbox version 4.10 (https://it.mathworks.com/products/mapping.html).

Fig 2A shows the epicenters of the mainshocks with magnitude larger than M3.7, occurred in Italy from 1973 to 2019, according to CPTI15. (Note that data associated to mainshock with magnitude lower than the minimum of the tested hazard model were neglected.) Fig 2B shows the location of the 143 recording stations considered, and the color of the marker provides the interval, in terms of number of years, including the operating time. In the legend, the number below each interval (*n*. *sts*) is the number of stations with operating time within that interval. Thus, for each station, the number of mainshocks occurred within 200 km during the operating time, based on CPTI15, is shown in Fig 2C. Finally, Fig 2D provides the number of mainshocks recorded by the stations according to ITACA data. Comparing Fig 2C and 2D reveals that not all the waveforms from CPTI15 mainshocks are available for the stations. This can be linked to the fact that stations may suffer of downtime due to, for example, breakdowns.

To account for this issue in the testing, the probability of a station missing a record was computed as the number of non-recorded mainshocks divided by the number of mainshocks occurred within 200 km (available from the seismic catalog). A marginal probability for all stations (equal to 0.09) was then computed. This is used in the test as discussed in section Hypothesis testing.

### Counting exceedances

**Fig 3:**
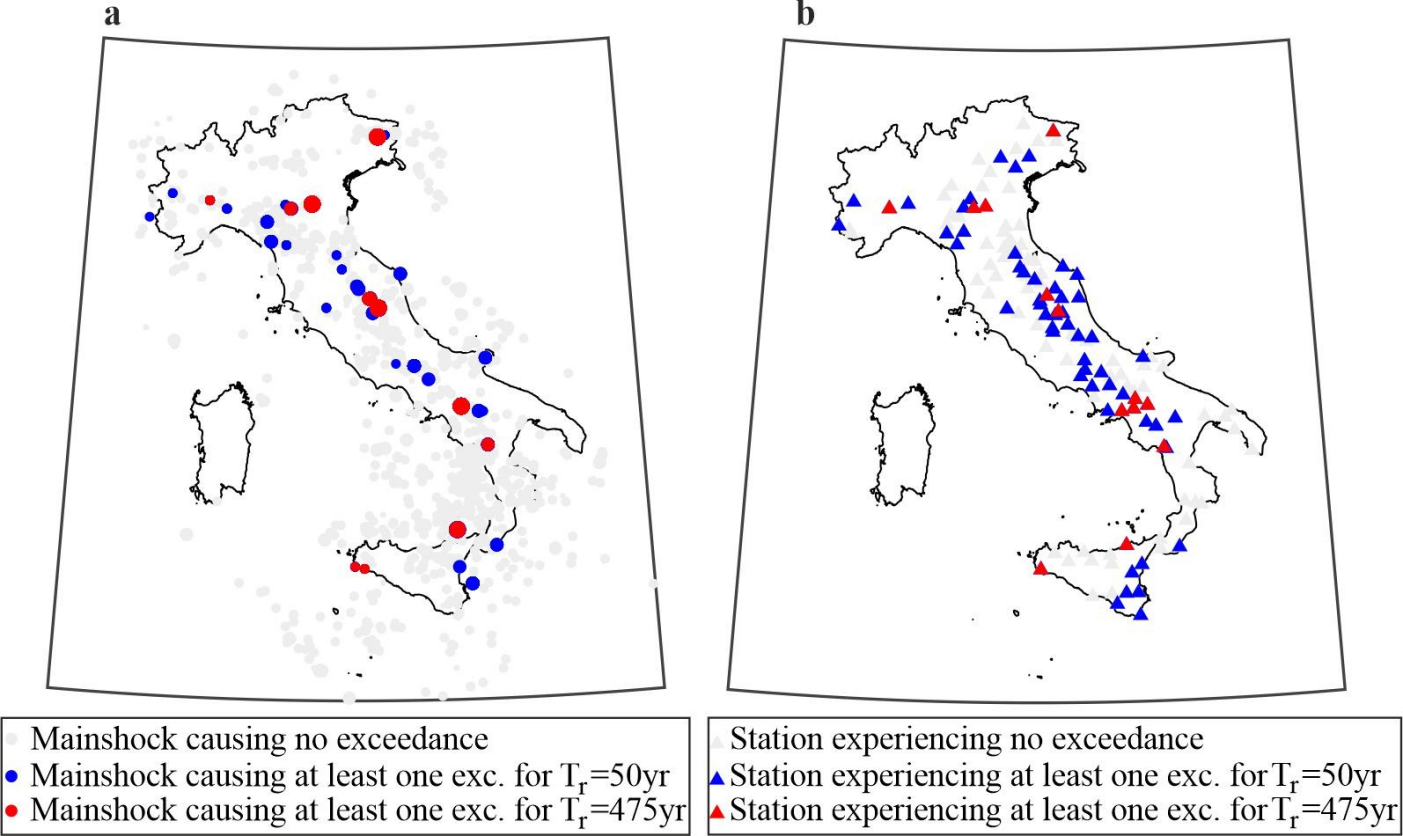
Mainshocks causing exceedance and stations experiencing exceedance. (a) Map of the M3.7+ mainshocks from 1973 to 2019 according to CPTI15: grey are those causing no exceedances at any station within 200 km from the epicenter; bluish and reddish are those causing exceedance of at least one ***IM*** (among the three considered) with ***T***_***r***_ = **50*yr*** and ***T***_***r***_ = **475*yr***, respectively. (b) Map of the stations experiencing no exceedance (grey) and those subjected to at least one exceedance of at least one ***IM*** with ***T***_***r***_ = **50*yr*** (bluish) and ***T***_***r***_ = **475*yr*** (reddish), for at least one hazard model. The maps were generated using the Matlab mapping toolbox version 4.10 (https://it.mathworks.com/products/mapping.html).

Comparing the dataset with the hazard associated intensity thresholds from the different hazard maps (for different return periods and *IMs*) at the sites of the stations (see S2 Table), it is possible to count the observed exceedances. The thresholds were adjusted for the site conditions of the stations via soil-specific amplification factors, provided by the GMMs [37]. As an example, Fig 3A maps, with grey marks, the mainshocks from the CPTI15 (i.e., those in Fig 2A), occurring within 200 km from at least one recording station, yet not causing the exceedance of none of the considered *IM* thresholds when *T*_*r*_ = 50*yr*, for any hazard model. The bluish and reddish dots are the mainshocks causing exceedance, at least at one station, of at least one *IM* (among those considered herein) and hazard model, when the exceedance thresholds correspond to *T*_*r*_ = 50*yr* and *T*_*r*_ = 475*yr*, respectively.

The stations recording at least one exceedance, in the case of at least one *IM*, for at least one hazard model, during their operating time, are mapped in Fig 3B. The grey ones are those experiencing zero exceedances, whereas the bluish and reddish refer to *T*_*r*_ = 50*yr* and *T*_*r*_ = 475*yr*, respectively. The figure shows that earthquakes causing exceedance, as well as stations subjected to at least one exceedance, are spread throughout the medium-to-high hazardous regions of the country such as the Apennine Mountain chain, northeast Italy and Sicily, as expected (see Fig 1). The figure shows that 39 mainshocks caused exceedance of at least one *IM* associated to at least one hazard model at 64 out of 143 stations.

**Fig 4:**
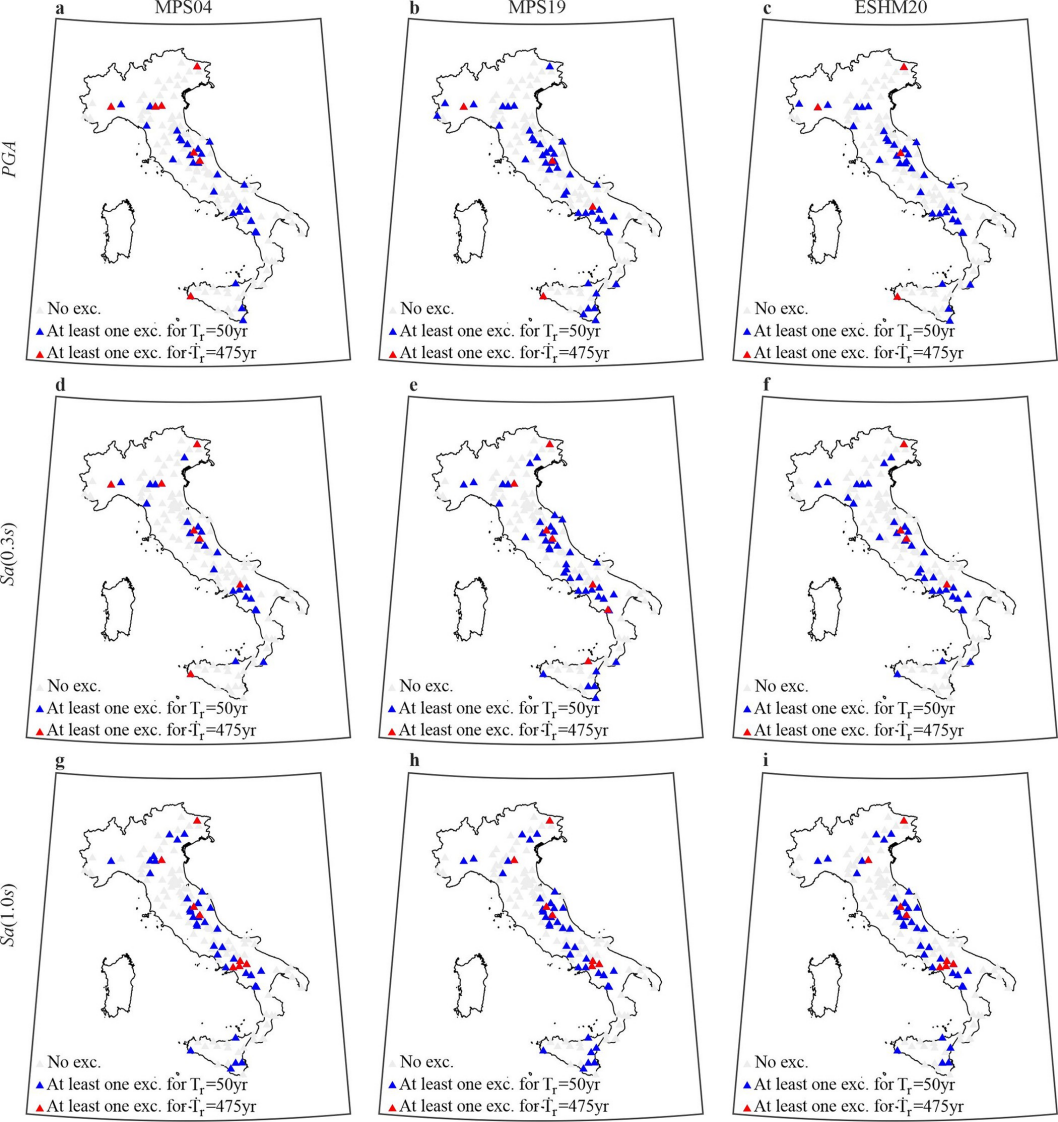
Stations experiencing exceedance. Maps of mainshock exceedance (and non-exceedance) at the stations of the ***IM*** thresholds with ***T***_***r***_ **= 50*yr*** and ***T***_***r***_ **= 475*yr***. The panels in the same columns refer to the same model: (a), (d), (g) are from MPS04; (b), (e), (h) are from MPS19; (c), (f), (i) are from ESHM20. The panels in the same row refer to the same ***IM***: (a), (b), (c) are ***PGA***; (d), (e), (f) are ***Sa***(**0.3*s***); (g), (h), (i) are ***Sa***(**1.0*s***). The maps were generated using the Matlab mapping toolbox version 4.10 (https://it.mathworks.com/products/mapping.html).

Fig 4 shows the stations experiencing at least one exceedance, considering the *IMs* and hazard models individually (the list of stations experiencing exceedance is given in S3–S5 Tables, referring to MPS04, MPS19 and ESHM20, respectively). Panels a-c refer to *PGA*, d-f to *Sa*(0.3*s*) and g-i *Sa*(1.0*s*). As in Fig 3B, stations not experiencing exceedance are marked in grey, whereas bluish and reddish correspond to threshold associated to *T*_*r*_ = 50*yr* and *T*_*r*_ = 475*yr*, respectively. For each *IM*, the geographic distribution of the stations experiencing at least one exceedance does not vary significantly with the considered hazard model.

### Hypothesis testing

For each station among those considered, the several-years-long seismic history (close to fifty years in some cases) was carefully scrutinized and the number of exceedances, caused by the identified mainshocks in the catalog, and with magnitude above the minimum of the considered model, were counted. Based on this information, for each hazard model (MPS04, MPS19, and ESHM20), each return period (50, 475, 975, and 2475 years), and each *IM* (*PGA*, *Sa*(0.3*s*), *Sa*(1.0*s*)) a hypothesis test was performed, resulting in a total of thirty-six tests. The hypothesis under testing is always that the hazard maps are compatible with the number of observed exceedances at 143 sites where seismic recording stations have been continuously operating between 1973 and 2019. The total number of exceedances collectively observed at the stations represents the realization of the test statistic. If such a value falls in the non-rejection region the test is passed. (Note that for each map the number of exceedances for the same return period and *IM* changes because the thresholds to compute exceedances are hazard-model-dependent).

To build the non-rejection region of the test, the probability distributions of the random variable (RV) counting the total number of exceedances at the 143 stations sites during their operating time was needed (one distribution for each tested map, that is, three hazard models by three *IM* and four return periods; see Table 1). Such a distribution was derived via multi-site probabilistic seismic hazard analysis [38], following the procedure theoretically justified in other studies [39] and recalled here for the purposes of this work. More specifically, several synthetic catalogs of earthquakes, 5∙10^5^ in number, occurring in Italy in 50 years periods were simulated. This period was selected to be sure to cover the operating time of the stations that worked the longest, yet exceedances were counted considering the effective operating time. In the *i*−*th* simulation, *i* = 1,…,5∙10^5^, a number of earthquakes, *n*_*i*_, was sampled from a Poisson distribution with annual rate equal to the sum of the annual rates of the earthquakes of the sources contemplated by the hazard model to be tested. The events occurrence time conditional to *n*_*i*_ (which is a uniform RV according to the Poisson process) was also simulated. Then, for the *j*−*th* earthquake of the *i*−*th* simulation, ∀*j* = 1,…,*n*_*i*_ and ∀*i* = 1,…,5∙10^5^, the considered *IM* was simulated at each of the 143 station sites, as follows.

1. The seismic source where the earthquake occurs is sampled, considering that the probability that an earthquake comes from one specific source is equal to the ratio between the earthquakes rate for that source and the sum of the rates of all the sources belonging to the hazard model.
2. The earthquake magnitude is sampled from the magnitude distribution for the source established at step (1), while the location of the earthquake epicenter is simulated considering that it is uniformly distributed over the source (the simulation of the epicenter is not needed in the case of the grid source model).
3. Given the earthquake magnitude and location, the *IM* realizations at the station locations are simulated accounting for the spatial correlation among GMM residuals at the different sites [40]; for each hazard model, the spatial correlation in ground motion is modelled according to Esposito and Iervolino [41].
4. The values resulting from step (3) are compared to the thresholds at the station sites with a certain return period from the hazard map, to mark eventual exceedance in the simulation.
5. For each station, the exceedance is not counted if the earthquake occurs out of its operating time, which is verified by comparing the earthquake occurrence time with the installation and removal years specific for the station. Also, the exceedance is not counted if the earthquake occurs when the station is operational, but it is not recording, which is simulated sampling from a Bernoulli RV with parameter equal to 0.09 (to account for non-recorded mainshocks).
6. The number of exceedances obtained for each earthquake *j* = 1,…,*n*_*i*_ are summed to get the number of exceedances for the *i*−*th* simulation, *i* = 1,…,5∙10^5^. Finally, the probability that the number of exceedances of the thresholds, in terms of the considered *IM* with return period established at step (4), is equal to *k*, is given by the number of simulations in which *k* exceedances are found divided by 5∙10^5^.

**Table 1:** Comparison between the non-rejection region and the number of exceedances collectively observed at the stations from 1973 to 2019 for intensity measure, return period, and hazard model.

	*T*_*r*_ = 50*yr*		
	MPS04	MPS19	ESHM20
***PGA***	46 ∉ [76–140]	55 ∉ [75–165]	48 ∉ [76–163]
***Sa*(0.3*s*)**	36 ∉ [71–145]	59 ∉ [67–174]	45 ∉ [74–164]
***Sa*(1.0*s*)**	52 ∉ [55–165]	54 ∈ [45–201]	51 ∉ [61–179]
	*T*_*r*_ = 475*yr*		
	**MPS04**	**MPS19**	**ESHM20**
***PGA***	8 ∈ [4–19]	4 ∈ [4–22]	4 ∉ [5–21]
***Sa*(0.3*s*)**	7 ∈ [4–20]	7 ∈ [3–25]	4 ∈ [4–21]
***Sa*(1.0*s*)**	8 ∈ [2–23]	7 ∈ [0–29]	8 ∈ [3–23]
	*T*_*r*_ = 975*yr*		
	**MPS04**	**MPS19**	**ESHM20**
***PGA***	4 ∈ [1–11]	1 ∈ [1–12]	0 ∉ [1–11]
***Sa*(0.3*s*)**	3 ∈ [1–11]	3 ∈ [0–13]	2 ∈ [1–12]
***Sa*(1.0*s*)**	2 ∈ [0–12]	2 ∈ [0–16]	1 ∈ [1–13]
	*T*_*r*_ = 2475*yr*		
	**MPS04**	**MPS19**	**ESHM20**
***PGA***	2 ∈ [0–5]	0 ∈ [0–6]	0 ∈ [0–5]
***Sa*(0.3*s*)**	1 ∈ [0–5]	1 ∈ [0–6]	0 ∈ [0–5]
***Sa*(1.0*s*)**	2 ∈ [0–6]	1 ∈ [0–7]	1 ∈ [0–6]

**Fig 5:**
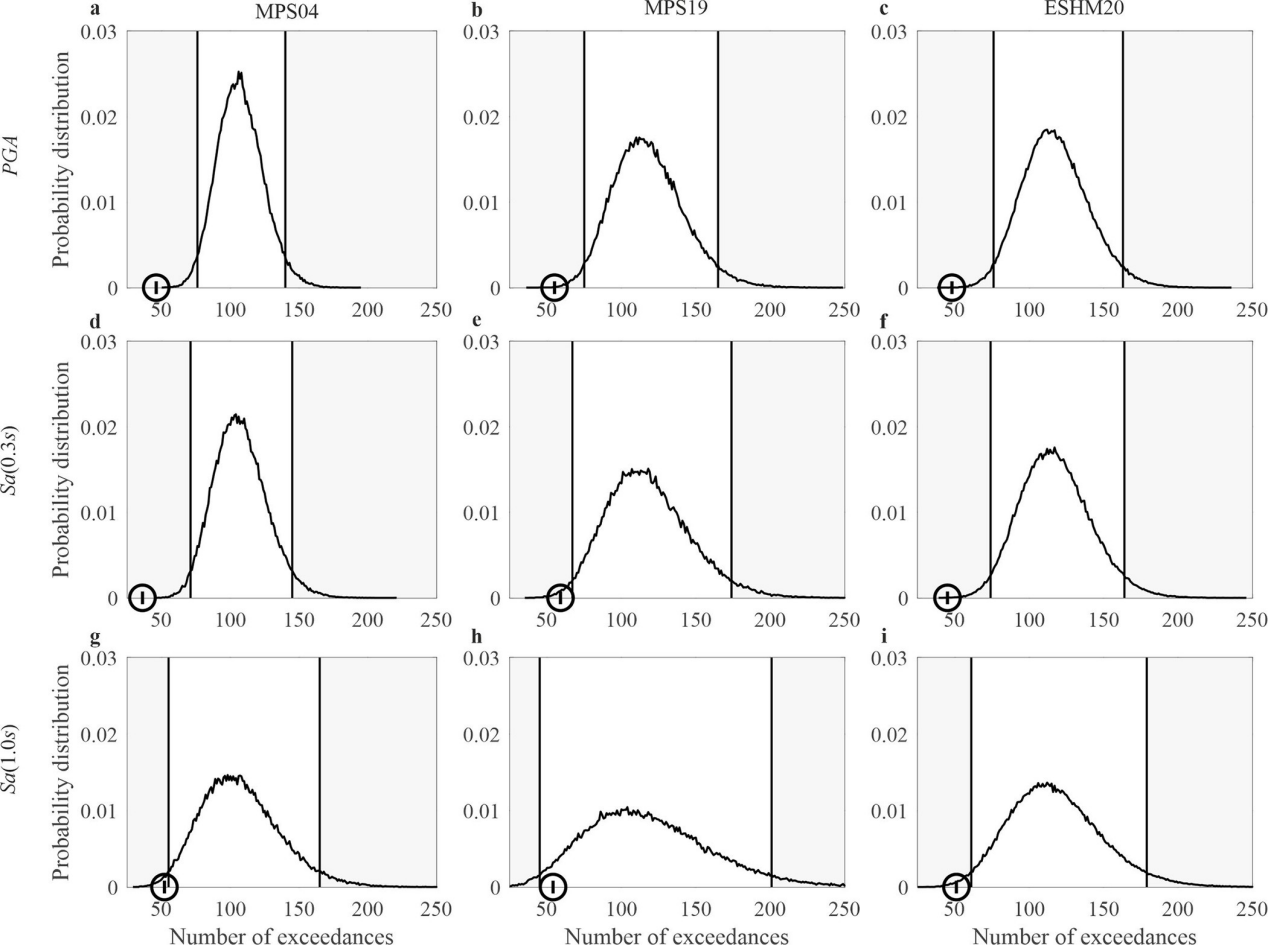
Results of the tests for *T*_*r*_ = 50*yr*. Distribution of the total number of exceedances from 1973 to 2019 of the ground motion intensity with *T*_*r*_ = 50*yr* at 143 sites where continuously recording stations have been present, each in a time period, and total observed exceedances at the same stations. The panels in the same columns refer to the same model: (a), (d), (g) are from MPS04; (b), (e), (h) are from MPS19; (c), (f), (i) are from ESHM20. The panels in the same row refer to the same ***IM***: (a), (b), (c) are ***PGA***; (d), (e), (f) are ***Sa*(0.3*s*)**; (g), (h), (i) are ***Sa*(1.0*s*)**.

**Fig 6:**
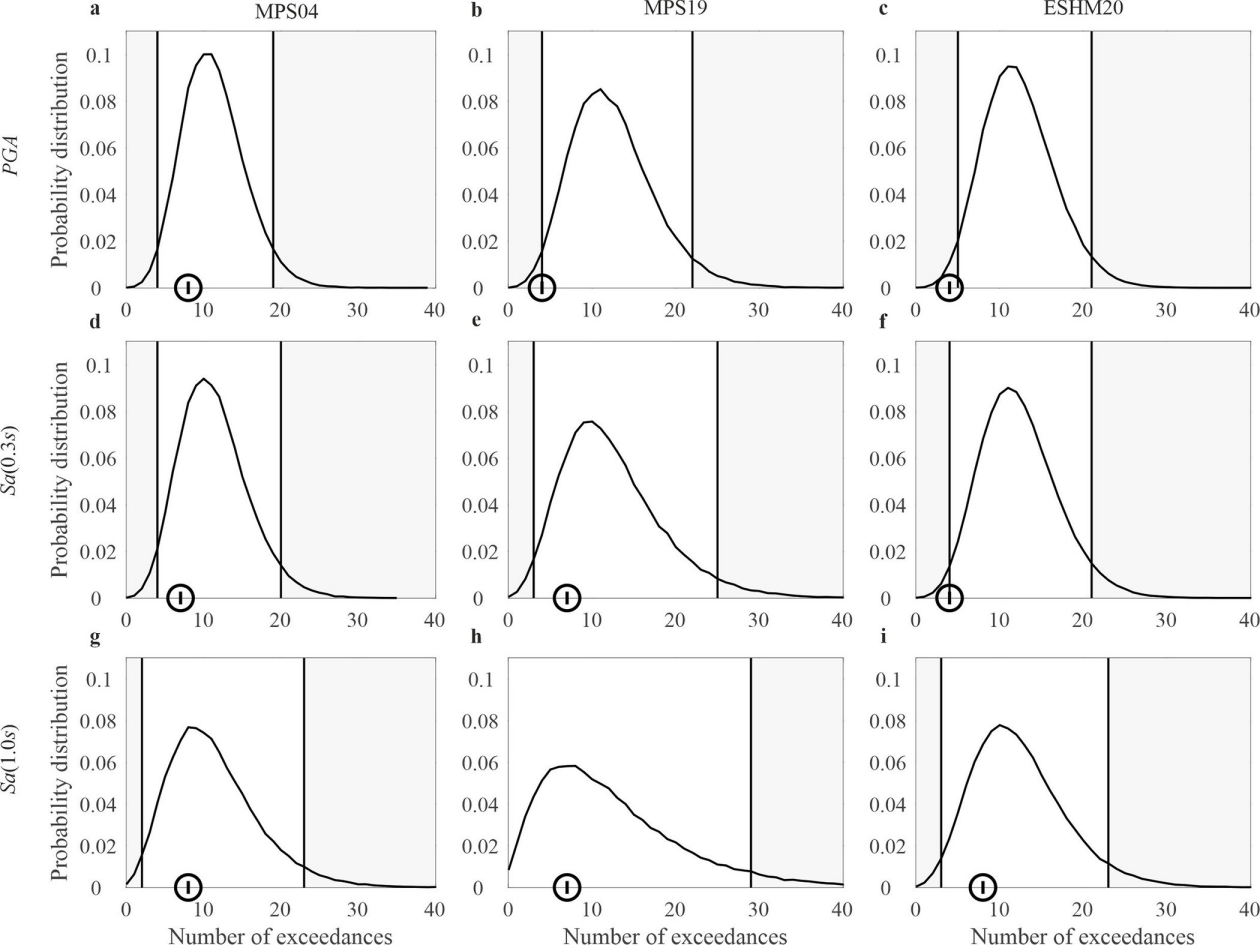
Results of the tests for *T*_*r*_ = 475*yr*. Distribution of the total number of exceedances from 1973 to 2019 of the ground motion intensity with *T*_*r*_ = 475*yr* at 143 sites where continuously recording stations have been present, each in a time period, and total observed exceedances at the same stations. The panels in the same columns refer to the same model: (a), (d), (g) are from MPS04; (b), (e), (h) are from MPS19; (c), (f), (i) are from ESHM20. The panels in the same row refer to the same ***IM***: (a), (b), (c) are ***PGA***; (d), (e), (f) are ***Sa*(0.3*s*)**; (g), (h), (i) are ***Sa*(1.0*s*)**.

The probabilities estimated in step (6) of the procedure were used to finally build the probability distributions of the total number of exceedances shown, as examples, in Figs 5 and 6. For each distribution, the lower and upper limits of the non-rejection region were obtained by excluding the realizations of the RV, starting from the tails, with increasing probability, until the sum of the probabilities associated to the removed realizations reaches 0.05, this one being an arbitrary, yet typical, significance level for hypothesis testing.

## Results

As an example of the test, Fig 5 shows the distributions of the number of exceedances at the station sites from 1973 to 2019 and the limits of the rejection regions, considering *T*_*r*_ = 50*yr* and the three *IMs* for the three hazard models. For each distribution, the number of recorded exceedances is marked with a circle. The maps pass the test only in the case of MPS19 and the *Sa*(1.0*s*). It is also to note that, for all models, the empirical data are in the left tail of the distribution, that is, below the expected value. Fig 6 shows the same results as Fig 5, yet for *T*_*r*_ = 475*yr*. For *Sa*(0.3*s*) and *Sa*(1.0*s*), all the maps pass the test, except ESHM20 in the case of *PGA*.

A comprehensive picture of the results is given in Table 1, which reports all the four return periods and three *IMs* considered to test the three hazard models for Italy. Each cell provides the number of counted exceedances for the case under examination and the interval representing the non-rejection region. To identify the trend of the results at a glance, failure is reported in red and pass in green.

It appears that all the three PSHA models are comparable, if not essentially equivalent, with respect to the test results, in the sense that, in nine out of twelve pairs of return period and *IM*, the test result does not vary with the hazard model considered. The null hypothesis is rejected almost always when *T*_*r*_ = 50*yr* is concerned, with the only non-rejection occurring in the case of MPS19 model at *Sa*(1.0*s*) (as anticipated by Fig 5), whereas the opposite is found for *T*_*r*_ = 475*yr*, where the only failure occurs at *PGA* for ESHM20. A similar situation occurs when the considered return period is *T*_*r*_ = 975*yr*. Nevertheless, for both return period cases, the number of observed exceedances is close to the lower limit of the non-rejection region. Finally, when *T*_*r*_ = 2475*yr* no rejection occurs. (It is also worth noting that, instead of comparing test results for one return period and *IM*, the total number of rejections per hazard model could be compared adjusting the significance level accounting for the total number of tests as 0.05/12, according to the Bonferroni correction [42]).

It is not within the scope of this study, which refers to the main interest of earthquake engineering, that is PSHA output, to investigate the causes of these results in terms of the PSHA input models. However, it may be worthwhile to recall that the abundance of data at the lower return periods typically leads to easier rejection of formal hypothesis tests, which is a known issue in statistics. For the very same reason, the intensity thresholds corresponding to larger return periods are relatively rare to be exceeded, and therefore the number of observed exceedances is relatively low, such that the test cannot easily lead to failure. Morevoer, the apparent overestimation of hazard at *T*_*r*_ = 50*yr* can be due, for example, to the effect of the type of declustering of seismic catalogs, which affects the relative distribution of earthquake magnitudes [43]. Also, some residual issue related to completeness of recorded exceedances may still be present, despite what done in the study tried to avoid them. Interested researchers can investigate the roots of these results in the input models, as the study and the supporting information were carefully designed to be fully reproducible.

## Conclusions

Hazard maps for Italy, from three different authoritative PSHA studies (MPS04, MPS19, ESHM20), were compared testing whether exceedances of selected ground motion intensity measures, pooled from 143 seismic monitoring stations operating in about fifty years, are compatible with the distribution of the total number of exceedances at the sites, as derived from each of the hazard models. This was carried out considering selected ground motion intensity measures and return periods. Considering a 0.05 significance level the test showed that all the PSHA models perform comparably, except some cases, despite the apparent differences in the hazard maps. As a side result, it was found that that all models tend to overpredict exceedances at the lowest return period considered, while are compatible with exceedances recorded at the loner return periods. While the latter result is expected given the obvious relative paucity of exceedances for the rarest intensity threshold, the former is less so, yet it can be affected by some issues, discussed in the paper, in gathering the sample used to test the models.

## Supporting information

S1 Table Earthquake catalog. Earthquake catalog containing mainshocks up to 2019 considered in this study.
(XLSX)

S2 Table List of the stations considered in the study. The table provides, for each station, the coordinates, the soil class (according to Eurocode 8 classification) and the hazard threshold for each intensity measure, exceedance return period and hazard model.
(XLSX)

S3 Table Exceedances of MPS04 thresholds. Details of the exceedances, at the station sites, of the ground motion intensity measure thresholds associated to MPS04.
(XLSX)

S4 Table Exceedances of MPS19 thresholds. Details of the exceedances, at the station sites, of the ground motion intensity measure thresholds associated to MPS19.
(XLSX)

S5 Table Exceedances of ESHM20 thresholds. Details of the exceedances, at the station sites, of the ground motion intensity measure thresholds associated to ESHM20.
(XLSX)
